# Supplementary material for: Optimizing phage-antibiotic combinations: impact of administration order against daptomycin non-susceptible (DNS) MRSA clinical isolates
Source: Antimicrob Agents Chemother. 2025 Nov 18;69(12):e00699-25. doi: 10.1128/aac.00699-25 (PMC12691696; doi:10.1128/aac.00699-25)
Supplement: Fig. S1 — Pairwise comparisons of continuous growth suppression dynamics at time point 0h, against DNS-MRSA isolates. [file aac.00699-25-s0001.pdf]

## Supplementary Figure 1. (S1)

## Timepoint: Hour 0 (T0)

| Tukey's multiple comparisons test | Mean Diff. | 95.00% CI of diff. | Below threshold? | Summary | Adjusted P Value |
|---|---|---|---|---|---|
| C18 vs. 684 | -0.013 | -0.9063 to 0.8803 | No | ns | >0.9999 |
| C18 vs. C6 | -0.005 | -0.2683 to 0.2583 | No | ns | 0.9991 |
| C18 vs. JKD6005 | -0.0035 | -0.2846 to 0.2776 | No | ns | >0.9999 |
| C18 vs. C2 | -0.004 | -0.2504 to 0.2424 | No | ns | >0.9999 |
| C18 vs. C27 | -0.014 | -0.1665 to 0.1385 | No | ns | 0.8783 |
| C18 vs. C51 | -0.0005 | -0.1348 to 0.1338 | No | ns | >0.9999 |
| C18 vs. C8 | -0.003 | -0.1562 to 0.1502 | No | ns | >0.9999 |
| C18 vs. C39 | -0.013 | -0.1404 to 0.1144 | No | ns | 0.9725 |
| C18 vs. C31 | -0.0075 | -0.2260 to 0.2110 | No | ns | 0.9903 |
| C18 vs. C21 | -0.0095 | -0.2280 to 0.2090 | No | ns | 0.9657 |
| C18 vs. J03 | -0.0065 | -0.1444 to 0.1314 | No | ns | >0.9999 |
| C18 vs. C49 | -0.0025 | -0.2210 to 0.2160 | No | ns | >0.9999 |
| C18 vs. C43 | -0.0145 | -0.1870 to 0.1580 | No | ns | 0.9802 |
| C18 vs. C16 | -0.007 | -0.1595 to 0.1455 | No | ns | 0.9964 |
| C18 vs. C25 | -0.01 | -1.141 to 1.121 | No | ns | >0.9999 |
| 684 vs. C6 | 0.008 | -0.9898 to 1.006 | No | ns | >0.9999 |
| 684 vs. JKD6005 | 0.0095 | -0.9933 to 1.012 | No | ns | >0.9999 |
| 684 vs. C2 | 0.009 | -0.5248 to 0.5428 | No | ns | >0.9999 |
| 684 vs. C27 | -0.001 | -0.9788 to 0.9768 | No | ns | >0.9999 |
| 684 vs. C51 | 0.0125 | -0.9504 to 0.9754 | No | ns | >0.9999 |
| 684 vs. C8 | 0.01 | -0.7929 to 0.8129 | No | ns | >0.9999 |
| 684 vs. C39 | 0 | -0.8933 to 0.8933 | No | ns | >0.9999 |
| 684 vs. C31 | 0.0055 | -0.9839 to 0.9949 | No | ns | >0.9999 |
| 684 vs. C21 | 0.0035 | -0.9859 to 0.9929 | No | ns | >0.9999 |
| 684 vs. J03 | 0.0065 | -0.8496 to 0.8626 | No | ns | >0.9999 |
| 684 vs. C49 | 0.0105 | -0.9789 to 0.9999 | No | ns | >0.9999 |
| 684 vs. C43 | -0.0015 | -0.7148 to 0.7118 | No | ns | >0.9999 |
| 684 vs. C16 | 0.006 | -0.9718 to 0.9838 | No | ns | >0.9999 |
| 684 vs. C25 | 0.003 | -0.5162 to 0.5222 | No | ns | >0.9999 |
| C6 vs. JKD6005 | 0.0015 | -0.03663 to 0.03963 | No | ns | 0.9989 |
| C6 vs. C2 | 0.001 | -0.4879 to 0.4899 | No | ns | >0.9999 |
| C6 vs. C27 | -0.009 | -0.08526 to 0.06726 | No | ns | 0.7608 |
| C6 vs. C51 | 0.0045 | -0.1186 to 0.1276 | No | ns | 0.9932 |
| C6 vs. C8 | 0.002 | -0.3381 to 0.3421 | No | ns | >0.9999 |
| C6 vs. C39 | -0.008 | -0.2713 to 0.2553 | No | ns | 0.9803 |
| C6 vs. C31 | -0.0025 | -0.05127 to 0.04627 | No | ns | 0.999 |
| C6 vs. C21 | -0.0045 | -0.05327 to 0.04427 | No | ns | 0.9533 |
| C6 vs. J03 | -0.0015 | -0.3036 to 0.3006 | No | ns | >0.9999 |

| | | | | | |
|---|---|---|---|---|---|
| C6 vs. C49 | 0.0025 | -0.04627 to 0.05127 | No | ns | 0.999 |
| C6 vs. C43 | -0.0095 | -0.3872 to 0.3682 | No | ns | 0.9899 |
| C6 vs. C16 | -0.002 | -0.07826 to 0.07426 | No | ns | >0.9999 |
| C6 vs. C25 | -0.005 | -1.219 to 1.209 | No | ns | >0.9999 |
| JKD6005 vs. C2 | -0.0005 | -0.4994 to 0.4984 | No | ns | >0.9999 |
| JKD6005 vs. C27 | -0.0105 | -0.1422 to 0.1212 | No | ns | 0.6522 |
| JKD6005 vs. C51 | 0.003 | -0.1670 to 0.1730 | No | ns | 0.9993 |
| JKD6005 vs. C8 | 0.0005 | -0.3535 to 0.3545 | No | ns | >0.9999 |
| JKD6005 vs. C39 | -0.0095 | -0.2906 to 0.2716 | No | ns | 0.9491 |
| JKD6005 vs. C31 | -0.004 | -0.09408 to 0.08608 | No | ns | 0.9387 |
| JKD6005 vs. C21 | -0.006 | -0.09608 to 0.08408 | No | ns | 0.7926 |
| JKD6005 vs. J03 | -0.003 | -0.3207 to 0.3147 | No | ns | >0.9999 |
| JKD6005 vs. C49 | 0.001 | -0.08908 to 0.09108 | No | ns | >0.9999 |
| JKD6005 vs. C43 | -0.011 | -0.4013 to 0.3793 | No | ns | 0.9752 |
| JKD6005 vs. C16 | -0.0035 | -0.1352 to 0.1282 | No | ns | 0.9906 |
| JKD6005 vs. C25 | -0.0065 | -1.225 to 1.212 | No | ns | >0.9999 |
| C2 vs. C27 | -0.01 | -0.4566 to 0.4366 | No | ns | 0.9979 |
| C2 vs. C51 | 0.0035 | -0.3980 to 0.4050 | No | ns | >0.9999 |
| C2 vs. C8 | 0.001 | -0.2200 to 0.2220 | No | ns | >0.9999 |
| C2 vs. C39 | -0.009 | -0.2554 to 0.2374 | No | ns | 0.9998 |
| C2 vs. C31 | -0.0035 | -0.4757 to 0.4687 | No | ns | >0.9999 |
| C2 vs. C21 | -0.0055 | -0.4777 to 0.4667 | No | ns | >0.9999 |
| C2 vs. J03 | -0.0025 | -0.2341 to 0.2291 | No | ns | >0.9999 |
| C2 vs. C49 | 0.0015 | -0.4707 to 0.4737 | No | ns | >0.9999 |
| C2 vs. C43 | -0.0105 | -0.2255 to 0.2045 | No | ns | 0.9998 |
| C2 vs. C16 | -0.003 | -0.4496 to 0.4436 | No | ns | >0.9999 |
| C2 vs. C25 | -0.006 | -0.7984 to 0.7864 | No | ns | >0.9999 |
| C27 vs. C51 | 0.0135 | -0.06308 to 0.09008 | No | ns | 0.7294 |
| C27 vs. C8 | 0.011 | -0.2353 to 0.2573 | No | ns | 0.978 |
| C27 vs. C39 | 0.001 | -0.1515 to 0.1535 | No | ns | >0.9999 |
| C27 vs. C31 | 0.0065 | -0.05561 to 0.06861 | No | ns | 0.9459 |
| C27 vs. C21 | 0.0045 | -0.05761 to 0.06661 | No | ns | 0.9934 |
| C27 vs. J03 | 0.0075 | -0.1813 to 0.1963 | No | ns | 0.9967 |
| C27 vs. C49 | 0.0115 | -0.05061 to 0.07361 | No | ns | 0.6753 |
| C27 vs. C43 | -0.0005 | -0.3110 to 0.3100 | No | ns | >0.9999 |
| C27 vs. C16 | 0.007 | -0.05669 to 0.07069 | No | ns | 0.9594 |
| C27 vs. C25 | 0.004 | -1.194 to 1.202 | No | ns | >0.9999 |
| C51 vs. C8 | -0.0025 | -0.1931 to 0.1881 | No | ns | >0.9999 |
| C51 vs. C39 | -0.0125 | -0.1468 to 0.1218 | No | ns | 0.9376 |
| C51 vs. C31 | -0.007 | -0.09272 to 0.07872 | No | ns | 0.9593 |
| C51 vs. C21 | -0.009 | -0.09472 to 0.07672 | No | ns | 0.8869 |
| C51 vs. J03 | -0.006 | -0.1669 to 0.1549 | No | ns | 0.9997 |
| C51 vs. C49 | -0.002 | -0.08772 to 0.08372 | No | ns | >0.9999 |
| C51 vs. C43 | -0.014 | -0.2397 to 0.2117 | No | ns | 0.9612 |

| | | | | | |
|---|---|---|---|---|---|
| C51 vs. C16 | -0.0065 | -0.08308 to 0.07008 | No | ns | 0.986 |
| C51 vs. C25 | -0.0095 | -1.195 to 1.176 | No | ns | >0.9999 |
| C8 vs. C39 | -0.01 | -0.1632 to 0.1432 | No | ns | 0.9978 |
| C8 vs. C31 | -0.0045 | -0.3187 to 0.3097 | No | ns | >0.9999 |
| C8 vs. C21 | -0.0065 | -0.3207 to 0.3077 | No | ns | 0.999 |
| C8 vs. J03 | -0.0035 | -0.1571 to 0.1501 | No | ns | >0.9999 |
| C8 vs. C49 | 0.0005 | -0.3137 to 0.3147 | No | ns | >0.9999 |
| C8 vs. C43 | -0.0115 | -0.1808 to 0.1578 | No | ns | 0.9981 |
| C8 vs. C16 | -0.004 | -0.2503 to 0.2423 | No | ns | >0.9999 |
| C8 vs. C25 | -0.007 | -1.082 to 1.068 | No | ns | >0.9999 |
| C39 vs. C31 | 0.0055 | -0.2130 to 0.2240 | No | ns | 0.9989 |
| C39 vs. C21 | 0.0035 | -0.2150 to 0.2220 | No | ns | >0.9999 |
| C39 vs. J03 | 0.0065 | -0.1314 to 0.1444 | No | ns | >0.9999 |
| C39 vs. C49 | 0.0105 | -0.2080 to 0.2290 | No | ns | 0.9468 |
| C39 vs. C43 | -0.0015 | -0.1740 to 0.1710 | No | ns | >0.9999 |
| C39 vs. C16 | 0.006 | -0.1465 to 0.1585 | No | ns | 0.9989 |
| C39 vs. C25 | 0.003 | -1.128 to 1.134 | No | ns | >0.9999 |
| C31 vs. C21 | -0.002 | -0.04976 to 0.04576 | No | ns | >0.9999 |
| C31 vs. J03 | 0.001 | -0.2692 to 0.2712 | No | ns | >0.9999 |
| C31 vs. C49 | 0.005 | -0.04276 to 0.05276 | No | ns | 0.9685 |
| C31 vs. C43 | -0.007 | -0.3623 to 0.3483 | No | ns | 0.9991 |
| C31 vs. C16 | 0.0005 | -0.06161 to 0.06261 | No | ns | >0.9999 |
| C31 vs. C25 | -0.0025 | -1.210 to 1.205 | No | ns | >0.9999 |
| C21 vs. J03 | 0.003 | -0.2672 to 0.2732 | No | ns | >0.9999 |
| C21 vs. C49 | 0.007 | -0.04076 to 0.05476 | No | ns | 0.8649 |
| C21 vs. C43 | -0.005 | -0.3603 to 0.3503 | No | ns | >0.9999 |
| C21 vs. C16 | 0.0025 | -0.05961 to 0.06461 | No | ns | >0.9999 |
| C21 vs. C25 | -0.0005 | -1.208 to 1.207 | No | ns | >0.9999 |
| J03 vs. C49 | 0.004 | -0.2662 to 0.2742 | No | ns | >0.9999 |
| J03 vs. C43 | -0.008 | -0.1761 to 0.1601 | No | ns | 0.9999 |
| J03 vs. C16 | -0.0005 | -0.1893 to 0.1883 | No | ns | >0.9999 |
| J03 vs. C25 | -0.0035 | -1.109 to 1.102 | No | ns | >0.9999 |
| C49 vs. C43 | -0.012 | -0.3673 to 0.3433 | No | ns | 0.9711 |
| C49 vs. C16 | -0.0045 | -0.06661 to 0.05761 | No | ns | 0.9934 |
| C49 vs. C25 | -0.0075 | -1.215 to 1.200 | No | ns | >0.9999 |
| C43 vs. C16 | 0.0075 | -0.3030 to 0.3180 | No | ns | 0.9989 |
| C43 vs. C25 | 0.0045 | -1.032 to 1.041 | No | ns | >0.9999 |
