# Supplementary material for: Optimizing phage-antibiotic combinations: impact of administration order against daptomycin non-susceptible (DNS) MRSA clinical isolates
Source: Antimicrob Agents Chemother. 2025 Nov 18;69(12):e00699-25. doi: 10.1128/aac.00699-25 (PMC12691696; doi:10.1128/aac.00699-25)
Supplement: Fig. S2 — Pairwise comparisons of continuous growth suppression dynamics at time point 24h, against DNS-MRSA isolates exposed to subinhibitory concentrations of DAP and CPT (0.5x MIC). [file aac.00699-25-s0002.pdf]

Supplementary Figure 2. (S2)

Timepoint: Hour 24 (T24)

Tukey's multiple comparisons test	Mean Diff.	95.00% CI of diff.	Below threshold?	Summary	Adjusted P Value
C18 vs. 684	-1.032	-1.398 to -0.6646	Yes	**	0.0073
C18 vs. C6	-0.9715	-2.547 to 0.6043	No	ns	0.0744
C18 vs. JKD6005	-0.7045	-0.9945 to -0.4145	Yes	*	0.0119
C18 vs. C2	-0.8885	-1.436 to -0.3407	Yes	*	0.0165
C18 vs. C27	-0.584	-15.26 to 14.10	No	ns	0.901
C18 vs. C51	0.0115	-0.2450 to 0.2680	No	ns	0.9998
C18 vs. C8	-0.778	-2.726 to 1.170	No	ns	0.1292
C18 vs. C39	-0.202	-6.882 to 6.478	No	ns	0.9655
C18 vs. C31	-0.878	-1.126 to -0.6295	Yes	**	0.002
C18 vs. C21	-0.0625	-3.568 to 3.443	No	ns	0.9989
C18 vs. J03	-0.112	-0.7220 to 0.4980	No	ns	0.5113
C18 vs. C49	-1.002	-1.257 to -0.7473	Yes	**	0.0012
C18 vs. C43	-0.0635	-4.085 to 3.958	No	ns	0.9996
C18 vs. C16	-0.8545	-1.770 to 0.06054	No	ns	0.0524
C18 vs. C25	-0.9985	-7.643 to 5.646	No	ns	0.3561
684 vs. C6	0.06	-0.9944 to 1.114	No	ns	0.9733
684 vs. JKD6005	0.327	-0.02420 to 0.6782	No	ns	0.057
684 vs. C2	0.143	-0.6638 to 0.9498	No	ns	0.3051
684 vs. C27	0.4475	-14.20 to 15.10	No	ns	0.9625
684 vs. C51	1.043	0.5905 to 1.495	Yes	**	0.01
684 vs. C8	0.2535	-1.338 to 1.845	No	ns	0.419
684 vs. C39	0.8295	-5.783 to 7.442	No	ns	0.4222
684 vs. C31	0.1535	-0.2732 to 0.5802	No	ns	0.2278
684 vs. C21	0.969	-2.409 to 4.347	No	ns	0.1861
684 vs. J03	0.9195	0.4156 to 1.423	Yes	*	0.0186
684 vs. C49	0.0295	-0.3374 to 0.3964	No	ns	0.9811
684 vs. C43	0.968	-2.943 to 4.879	No	ns	0.2181
684 vs. C16	0.177	-0.4978 to 0.8518	No	ns	0.3778
684 vs. C25	0.033	-6.543 to 6.609	No	ns	>0.9999
C6 vs. JKD6005	0.267	-1.137 to 1.671	No	ns	0.3316
C6 vs. C2	0.083	-1.738 to 1.904	No	ns	0.8568
C6 vs. C27	0.3875	-14.02 to 14.80	No	ns	0.9819
C6 vs. C51	0.983	-0.7357 to 2.702	No	ns	0.0867
C6 vs. C8	0.1935	-0.7223 to 1.109	No	ns	0.6291
C6 vs. C39	0.7695	-5.311 to 6.850	No	ns	0.4417
C6 vs. C31	0.0935	-1.603 to 1.790	No	ns	0.8203
C6 vs. C21	0.909	-1.004 to 2.822	No	ns	0.152

C6 vs. J03	0.8595	0.005363 to 1.714	Yes	*	0.0494
C6 vs. C49	-0.0305	-1.606 to 1.545	No	ns	0.9996
C6 vs. C43	0.908	-1.510 to 3.326	No	ns	0.1923
C6 vs. C16	0.117	-0.6709 to 0.9049	No	ns	0.8139
C6 vs. C25	-0.027	-6.067 to 6.013	No	ns	>0.9999
JKD6005 vs. C2	-0.184	-0.8435 to 0.4755	No	ns	0.1937
JKD6005 vs. C27	0.1205	-14.55 to 14.79	No	ns	>0.9999
JKD6005 vs. C51	0.716	0.3839 to 1.048	Yes	*	0.0171
JKD6005 vs. C8	-0.0735	-1.920 to 1.773	No	ns	0.9553
JKD6005 vs. C39	0.5025	-6.152 to 7.157	No	ns	0.6408
JKD6005 vs. C31	-0.1735	-0.4906 to 0.1436	No	ns	0.1336
JKD6005 vs. C21	0.642	-2.815 to 4.099	No	ns	0.2926
JKD6005 vs. J03	0.5925	0.04083 to 1.144	Yes	*	0.0438
JKD6005 vs. C49	-0.2975	-0.5875 to -0.007531	Yes	*	0.0477
JKD6005 vs. C43	0.641	-3.338 to 4.620	No	ns	0.3358
JKD6005 vs. C16	-0.15	-0.9069 to 0.6069	No	ns	0.4606
JKD6005 vs. C25	-0.294	-6.912 to 6.324	No	ns	0.8706
C2 vs. C27	0.3045	-14.40 to 15.01	No	ns	0.9951
C2 vs. C51	0.9	0.5447 to 1.255	Yes	*	0.0117
C2 vs. C8	0.1105	-2.035 to 2.256	No	ns	0.8083
C2 vs. C39	0.6865	-6.056 to 7.429	No	ns	0.5014
C2 vs. C31	0.0105	-0.3845 to 0.4055	No	ns	0.9906
C2 vs. C21	0.826	-2.793 to 4.445	No	ns	0.2391
C2 vs. J03	0.7765	-0.3583 to 1.911	No	ns	0.0874
C2 vs. C49	-0.1135	-0.6613 to 0.4343	No	ns	0.2619
C2 vs. C43	0.825	-3.297 to 4.947	No	ns	0.2713
C2 vs. C16	0.034	-1.354 to 1.422	No	ns	0.9877
C2 vs. C25	-0.11	-6.817 to 6.597	No	ns	0.9991
C27 vs. C51	0.5955	-14.10 to 15.29	No	ns	0.8948
C27 vs. C8	-0.194	-14.49 to 14.10	No	ns	0.9999
C27 vs. C39	0.382	-7.965 to 8.729	No	ns	0.9933
C27 vs. C31	-0.294	-14.99 to 14.40	No	ns	0.9962
C27 vs. C21	0.5215	-13.01 to 14.06	No	ns	0.9416
C27 vs. J03	0.472	-14.12 to 15.06	No	ns	0.9537
C27 vs. C49	-0.418	-15.10 to 14.26	No	ns	0.9721
C27 vs. C43	0.5205	-12.63 to 13.67	No	ns	0.9449
C27 vs. C16	-0.2705	-14.80 to 14.26	No	ns	0.998
C27 vs. C25	-0.4145	-8.802 to 7.973	No	ns	0.9887
C51 vs. C8	-0.7895	-2.848 to 1.269	No	ns	0.1388
C51 vs. C39	-0.2135	-6.928 to 6.501	No	ns	0.9558
C51 vs. C31	-0.8895	-1.074 to -0.7045	Yes	***	0.0001
C51 vs. C21	-0.074	-3.641 to 3.493	No	ns	0.9959
C51 vs. J03	-0.1235	-1.067 to 0.8200	No	ns	0.463
C51 vs. C49	-1.014	-1.270 to -0.7570	Yes	***	0.0008

C51 vs. C43	-0.075	-4.151 to 4.001	No	ns	0.9982
C51 vs. C16	-0.866	-2.115 to 0.3827	No	ns	0.0701
C51 vs. C25	-1.01	-7.688 to 5.668	No	ns	0.3535
C8 vs. C39	0.576	-5.191 to 6.343	No	ns	0.5762
C8 vs. C31	-0.1	-2.141 to 1.941	No	ns	0.8554
C8 vs. C21	0.7155	-1.025 to 2.456	No	ns	0.2017
C8 vs. J03	0.666	-0.4326 to 1.765	No	ns	0.1087
C8 vs. C49	-0.224	-2.172 to 1.724	No	ns	0.4825
C8 vs. C43	0.7145	-1.419 to 2.848	No	ns	0.2512
C8 vs. C16	-0.0765	-1.064 to 0.9108	No	ns	0.9786
C8 vs. C25	-0.2205	-5.943 to 5.502	No	ns	0.9625
C39 vs. C31	-0.676	-7.384 to 6.032	No	ns	0.5073
C39 vs. C21	0.1395	-3.288 to 3.567	No	ns	0.9992
C39 vs. J03	0.09	-6.401 to 6.581	No	ns	0.9999
C39 vs. C49	-0.8	-7.480 to 5.880	No	ns	0.438
C39 vs. C43	0.1385	-3.052 to 3.329	No	ns	0.9995
C39 vs. C16	-0.6525	-7.019 to 5.714	No	ns	0.5183
C39 vs. C25	-0.7965	-3.782 to 2.189	No	ns	0.493
C31 vs. C21	0.8155	-2.741 to 4.372	No	ns	0.237
C31 vs. J03	0.766	-0.1080 to 1.640	No	ns	0.0543
C31 vs. C49	-0.124	-0.3725 to 0.1245	No	ns	0.1689
C31 vs. C43	0.8145	-3.252 to 4.881	No	ns	0.2708
C31 vs. C16	0.0235	-1.191 to 1.238	No	ns	0.9995
C31 vs. C25	-0.1205	-6.793 to 6.552	No	ns	0.9983
C21 vs. J03	-0.0495	-3.157 to 3.058	No	ns	>0.9999
C21 vs. C49	-0.9395	-4.445 to 2.566	No	ns	0.2017
C21 vs. C43	-0.001	-1.760 to 1.758	No	ns	>0.9999
C21 vs. C16	-0.792	-3.441 to 1.857	No	ns	0.2039
C21 vs. C25	-0.936	-4.335 to 2.463	No	ns	0.3333
J03 vs. C49	-0.89	-1.500 to -0.2800	Yes	*	0.0347
J03 vs. C43	0.0485	-3.647 to 3.744	No	ns	>0.9999
J03 vs. C16	-0.7425	-1.338 to -0.1467	Yes	*	0.0328
J03 vs. C25	-0.8865	-7.340 to 5.567	No	ns	0.3917
C49 vs. C43	0.9385	-3.083 to 4.960	No	ns	0.2326
C49 vs. C16	0.1475	-0.7675 to 1.063	No	ns	0.4699
C49 vs. C25	0.0035	-6.641 to 6.648	No	ns	>0.9999
C43 vs. C16	-0.791	-4.210 to 2.628	No	ns	0.2471
C43 vs. C25	-0.935	-4.099 to 2.229	No	ns	0.3319
