# Supplementary material for: Optimizing phage-antibiotic combinations: impact of administration order against daptomycin non-susceptible (DNS) MRSA clinical isolates
Source: Antimicrob Agents Chemother. 2025 Nov 18;69(12):e00699-25. doi: 10.1128/aac.00699-25 (PMC12691696; doi:10.1128/aac.00699-25)
Supplement: Fig. S3 — All pairwise comparisons in 24-hour time-kill analyses (antibiotics alone) against DNS-MRSA isolates exposed to subinhibitory concentrations of DAP and/or CPT at either 0.5x, 1x or 2x MIC. [file aac.00699-25-s0003.docx]

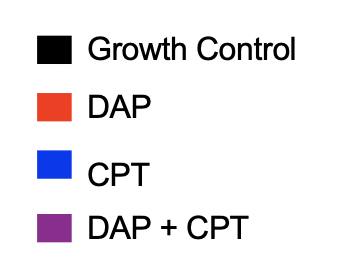


**Supplementary Figure S3.** All pairwise comparisons in 24h-hour time-kill analyses (antibiotics alone) against DNS-MRSA isolates exposed to subinhibitory concentrations of daptomycin (DAP) and/or ceftaroline (CPT) at either 0.5x, 1x or 2x MIC
