# Supplementary material for: Optimizing phage-antibiotic combinations: impact of administration order against daptomycin non-susceptible (DNS) MRSA clinical isolates
Source: Antimicrob Agents Chemother. 2025 Nov 18;69(12):e00699-25. doi: 10.1128/aac.00699-25 (PMC12691696; doi:10.1128/aac.00699-25)
Supplement: Fig. S6 — 24h-hour time-kill analyses assessing the use of 2 versus 3 phages in varying sequential administration sequences against strain C6. [file aac.00699-25-s0006.docx]

**Supplementary Figure S6.** 24h-hour time-kill analyses assessing the use of 2 versus 3 phages in varying sequential administration sequences against strain C6. Intesti13 + Sb-1 *2 phage cocktail; Intesti13 + Sb-1 + Romulus *3 phage cocktail; MOI_INPUT_ 1.0 PFU/mL; *DAP: daptomycin; CPT: ceftaroline; Φ: phage*
